# Searching for Low Molecular Weight Seleno-Compounds in Sprouts by Mass Spectrometry

**DOI:** 10.3390/molecules25122870

**Published:** 2020-06-22

**Authors:** Eliza Kurek, Magdalena Michalska-Kacymirow, Anna Konopka, Olga Kościuczuk, Anna Tomiak, Ewa Bulska

**Affiliations:** Faculty of Chemistry, Biological and Chemical Research Centre, University of Warsaw, Żwirki i Wigury 101, 02-089 Warsaw, Poland; ekurek@cnbc.uw.edu.pl (E.K.); m.kacymirow@cnbc.uw.edu.pl (M.M.-K.); a.konopka@cnbc.uw.edu.pl (A.K.); o.kosciuczuk@cnbc.uw.edu.pl (O.K.); atomiak@cnbc.uw.edu.pl (A.T.)

**Keywords:** selenium speciation, biotransformation of selenium in plants, low molecular weight seleno-compounds, mass spectrometry

## Abstract

A fit for purpose analytical protocol was designed towards searching for low molecular weight seleno-compounds in sprouts. Complementary analytical techniques were used to collect information enabling the characterization of selenium speciation. Conceiving the overall characterization of the behavior of selenium, inductively plasma optical mass spectrometry (ICP-MS) was used to determine the total selenium content in entire sprouts as well as in selected extracts or chromatographic fractions. Then, high-performance liquid chromatography combined with ICP-MS (HPLC-ICP-MS) was used to evaluate the presence of inorganic and organic seleno-compounds, with the advantages of being very sensitive towards selenium, but limited by available selenium standard compounds. Finally, ultra-high performance liquid chromatography electrospray ionization triple quadrupole mass spectrometry (UHPLC-ESI-QqQ-MS/MS) and UHPLC-ESI-Orbitrap-MS/MS were used for the confirmation of the identity of selected compounds and identification of several unknown compounds of selenium in vegetable sprouts (sunflower, onion, radish), respectively. Cultivation of plants was designed to supplement sprouts with selenium by using solutions of selenium (IV) at the concentration of 10, 20, 40, and 60 mg/L. The applied methodology allowed to justify that vegetable sprouts metabolize inorganic selenium to a number of organic derivatives, such as seleno-methylselenocysteine (SeMetSeCys), selenomethionine (SeMet), 5′-seleno-adenosine, 2,3-DHP-selenolanthionine, Se-S conjugate of cysteine-selenoglutathione, 2,3-DHP-selenocysteine-cysteine, 2,3-DHP-selenocysteine-cysteinealanine, glutathione-2,3-DHP-selenocysteine, gamma-Glu-MetSeCys or glutamyl-glycinyl-N-2,3-DHP-selenocysteine.

## 1. Introduction

Speciation studies are commonly conducted towards chemical characterization of various objects in respect to the chemical form of the element of interest [1]. In this work, we aim to evaluate the analytical performance of the developed approach combining complementary analytical techniques used to collect information enabling the broad characterization of selenium speciation in plants.

In general, preliminary characterization of the object involves the determination of the content of the element of interest [2], which is performed mainly by elemental techniques, such as atomic absorption spectrometry (ASA) [3], inductively plasma optical emission spectrometry (ICP-OES) or ICP mass spectrometry (ICP-MS) [4,5]. Then, the identification and quantification of selenium species require hyphenated methods. High-performance liquid chromatography combined with ICP-MS (HPLC-ICP-MS) is one of the most commonly used methods due to its high sensitivity towards selenium and tolerance to the sample matrix [6,7,8,9,10]. However, the complete characterization of the species of a given element is limited by available standards. Thus, the electrospray tandem mass spectrometry is used for the characterization of the compound of interest. A soft mode of electrospray ionization (ESI) enables to preserve the structure of the selenium molecule in order to determine its exact mass. Additionally, in MS/MS (tandem) mode, spectra of molecular fragments can be obtained, which are the base for elucidation of the entire molecule structure [11,12,13,14]. The use of the multiple reaction monitoring (MRM) mode in a triple quadrupole mass spectrometry (QqQ-MS/MS) system allows achieving a very good sensitivity for the identified compounds. In recent years, the advantages of an electrospray mass spectrometry equipped with a high-resolution mass analyzer Orbitrap, characterized by very high *m*/*z* measurement accuracy (below 1 mg/kg), high resolving power, and good sensitivity were explored in the case of unknown compounds for non-target analysis [12,15,16]. Thus, the use of a combined analytical approach becomes a powerful tool towards studying new selenium compounds and their metabolic pathways in plants.

For this study selenium was selected as it is considered to be of great importance for the proper functioning of the human organism with multiple biological activities, which depends on the content of selenium as well as the nature of the seleno-compounds [17]. The critical issue is a very narrow safety range between its deficiency and toxicity [18]. The level of selenium in the environment is very important, for example, in water, plants, and soil. The environmental levels of selenium (Se) are regulated and strictly controlled in the USA by the Environmental Protection Agency (EPA). The correlation between selenium and other elements is also important [19].

It is known that the effect of selenium depends not only on its concentration, but also its chemical forms, as they are subjected to various metabolic processes, and differ in bioavailability and toxicity [20,21,22].

The inorganic compounds are mostly selenite and selenate, while the organic compounds are selenoamino acids, selenopeptides, and selenoproteins [20,23]. Compared with inorganic selenium species, organic selenium compounds exhibit higher bioavailability, can be more readily absorbed, and are less toxic [24,25]. Organic compounds of selenium include low-molecular-weight compounds, such as selenium-containing derivatives of amino acids, such as selenomethionine (SeMet), seleno-methylselenocysteine (SeMetSeCys), L-γ-glutamyl-Se-methylseleno-L-cysteine (gamma-Glu-MetSeCys), but also proteins like selenoproteins containing selenium in the form of selenocysteine (Sec), like glutathione peroxidase, thioredoxin reductase, and selenoprotein P [6,7,8].

Selenoproteins are involved in redox metabolism and protect the tissues from the oxidative stress by catalyzing the reduction of peroxides and other oxidizing compounds. Oxidative stress is believed to be one of the key factors in the cancerogenesis, thus beneficial effects of selenium supplementation in cancer therapy can be expected. Although selenomethionine is the most studied organic selenium compound, it would be very valuable to identify other selenium compounds present in plants, which could be effective in cancer prevention. Recently, it was considered that monomethylated seleno-compounds, such as methyl-selenocysteine, could be potential candidates and they were chosen for clinical selenium supplementation trial of the National Cancer Institute (ClinicalTrials.gov; NCT01497431) [26]. Methyl-selenocysteine is transformed via γ-lyase reaction into methylselenol (CH_3_SeH), which is thought to be a key metabolite in the selenium anticancer properties [25,27]. It was suggested that cancer-preventing properties of seleno-compounds might depend on their conversion rate into CH_3_SeH [25,28].

The monomethylated selenium compounds have been identified in various selenium-enriched vegetables, such as garlic, onion [29], broccoli [30,31], green bean [30], and in the sprouts of various species [32]. Anticancer and antioxidant properties of these Se-enriched vegetables have also been evaluated [10,33,34,35,36,37,38].

Selenium biotransformation in plants very often follows the metabolic pathway of sulfur, due to their physicochemical similarity. But there are some exceptions specific for particular species, for example, by Indian mustard both inorganic and organic selenium compounds are metabolized to selenohomolanthionine [39]. SeMet behaves like methionine in the metabolic pathway (e.g., it can replace methionine in proteins), however the distributions of Se compounds can be quite different compared to that of their sulfur analogues [40,41]. Sprouts are expected to be a potential natural source of selenium compounds because they are a source of sulfur [42]. However, selenium (VI) ions compete with sulfate ions for access to membrane transporters, while phosphate ions may have an inhibitory effect on selenium (IV) uptake. This may indicate the use of phosphorus transport routes in the transport of Se (IV) [43]. Some works confirm good accumulation and biotransformation of selenium by selected types of sprouts [44,45,46,47].

Our work aimed to validate the analytical protocol involving mass spectrometry techniques to study selenium biotransformation by sprouts and to apply this protocol to evaluate the total content of selenium as well as to identify known, and characterize unknown, selenium compounds in selected plants of nutrition capability. The onion sprouts were chosen on the basis of our preliminary results, which indicated that onion metabolizes selenium compounds efficiently; not only to SeMet but also to SeMetSeCys [48] The Se-enriched sprouts were cultivated in hydroponics support supplemented with selenium selenite. The characterization of selenium compounds found in sprouts was performed by ICP-MS, HPLC-ICP-MS, ultra-high performance liquid chromatography electrospray ionization QqQ mass spectrometry (UHPLC-ESI-QqQ-MS/MS), and UHPLC-ESI-Orbitrap-MS/MS.

## 2. Results and Discussion

### 2.1. Total Selenium Concentration in Se-Enriched Sprouts

For the preliminary characteristic of the investigated sprouts, the total content of selenium in lyophilized sprout was determined (Table 1) by ICP-MS, monitoring ^82^Se isotope.

Limit of quantification (LOQ) and limit of detection (LOD) of selenium in dry weight of plant, for the entire procedure was estimated as 0.480 mg/kg and 0.356 mg/kg, respectively. Interestingly, all investigated sprouts exhibited good accumulation performance; the highest concentration of selenium was found in onion sprouts, compared to sprouts of radish and sunflowers. As it will be discussed later, this is also a link to the variety of the biotransformation of selenite into various seleno-amino acids.

### 2.2. Selenium Species in the Sprouts

#### 2.2.1. Identification of Selenium Compounds in Sprout’s Extracts by HPLC-ICP-MS

**Figure 1:**
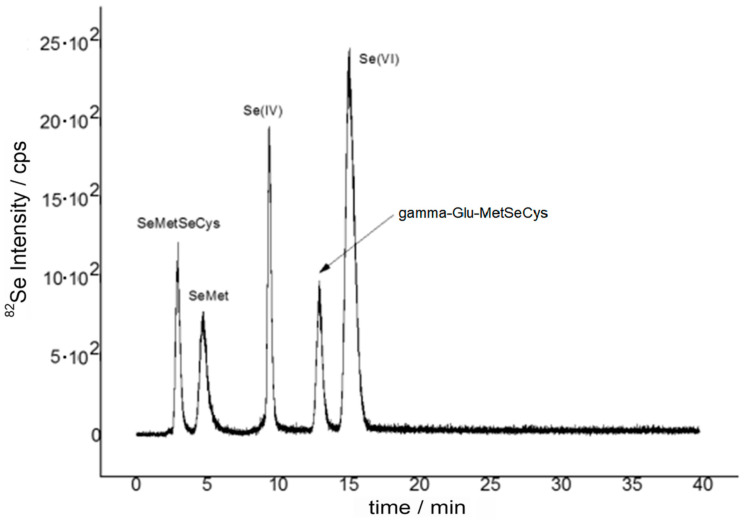
Chromatogram for standards: seleno-methylselenocysteine (SeMetSeCys), selenomethionine (SeMet), gamma-Glu-MetSeCys, selenite, and selenate with 500 µg/L of Se/L each, using anion exchange high-performance liquid chromatography (HPLC) coupled to inductively plasma optical mass spectrometry (ICP-MS); ^82^Se was monitored.

The commercially available selenium compounds were used to determine the retention times of anion exchange HPLC (Figure 1).

The solution of all available standards was prepared to contain 500 µg/L of selenium of each compound. As can be seen on the example chromatograms, signals appeared sequentially: (i) SeMetSeCys (2.8 min); (ii) Se-methionine (SeMet) (4.2 min); (iii) Se (IV) (9.1 min); (iv) gamma-Glu-MetSeCys (12.3 min); (v) Se (VI) (15.0 min) (Figure 1).

**Figure 2:**
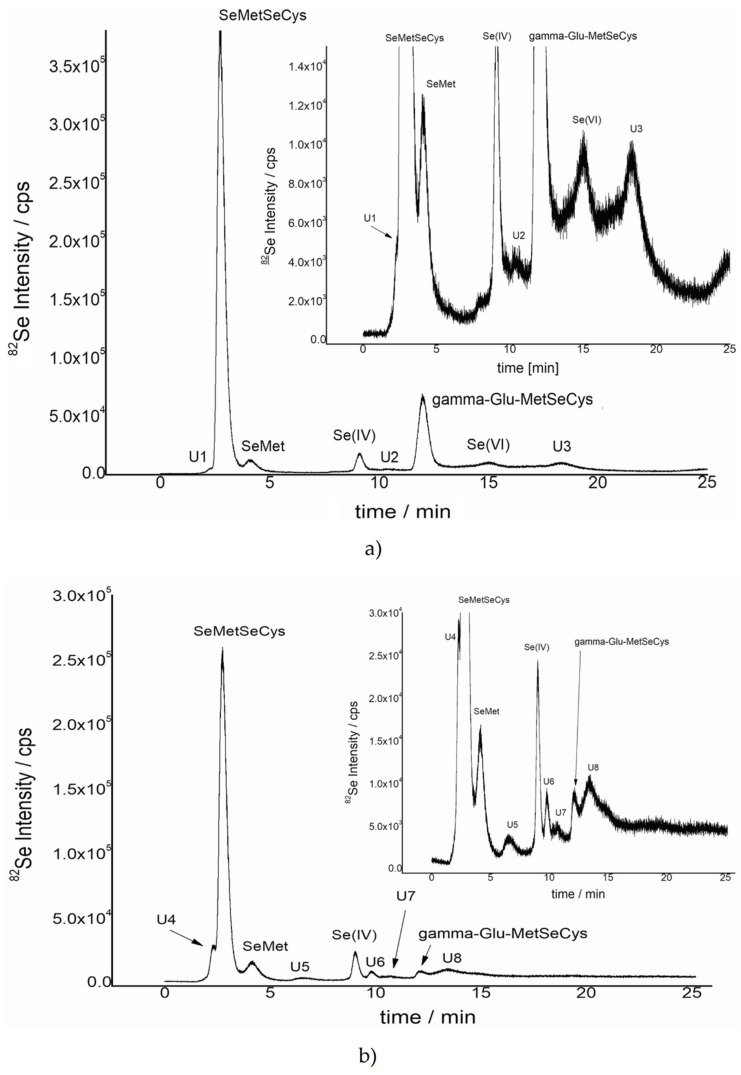

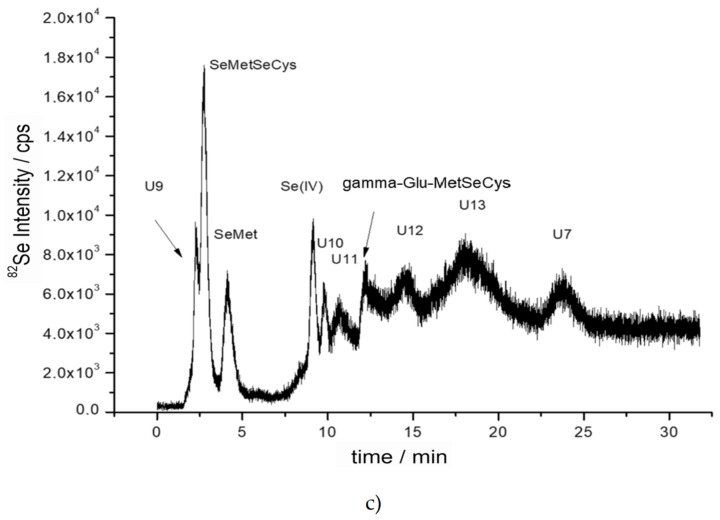
Chromatograms registered for extracts of (**a**) onion, (**b**) radish, and (**c**) sunflower sprouts, germinated in solutions containing 60 mg/L of Se as sodium selenite.

Selenium species were extracted from sprouts tissues using a mixture of protease and lipase in deionized water. Protease, which is responsible for the hydrolysis of peptide bonds, was expected to release selenium incorporated into the proteins in the form of selenoamino acids. The example chromatograms of selenium species enzymatically extracted from onion, radish, and sunflower sprouts germinated in solutions containing 60 mg/L of Se as sodium selenite are presented in Figure 2.

From obtained results is clear that the speciation of selenium depends on the type of sprouts. Some of the compounds are common for all sprouts, some of them are specific for a given type. Additionally, one can find some of the non-identified compounds, marked as U1 ÷ U13, all in different quantity.

Interestingly, SeMetSeCys was identified in all examined sprouts, but its relative occurrence strongly depends on the type of sprouts. The highest amount of SeMetSeCys and its derivative, gamma-Glu-MetSeCys, was found in onion sprouts. Moreover, the bigger content of selenite in the solution, the highest relative percentage of both compounds. In the case of radish and sunflower sprouts, the main compounds were those non-identified by HPLC-ICP-MS, above 60% and close to 80%, respectively. Thus, further experiments, focusing on the identification of those unknown compounds of selenium were conducted mainly on radish and sunflower sprouts.

#### 2.2.2. Identification of Selenium Compounds with the Use of Tandem Mass Spectrometry

To confirm the identity of selenium compounds detected by HPLC-ICP-MS, a targeted approach applying UHPLC-ESI-QqQ-MS/MS analysis, operating in multiple reaction monitoring (MRM) mode was employed. On the other hand, to identify the rest of non-identified seleno-compounds, a high resolution UHPLC-ESI-Orbitrap-MS/MS was used enabling a very accurate *m*/*z* registration (below 1 mg/kg) in a wide *m*/*z* range.

As described above, HPLC-ICP-MS allowed for determination of the presence of SeMet, SeMetSeCys, and gamma-Glu-MetSeCys. In Table 2, MRM transitions monitored for studied compounds are listed.

**Figure 3:**
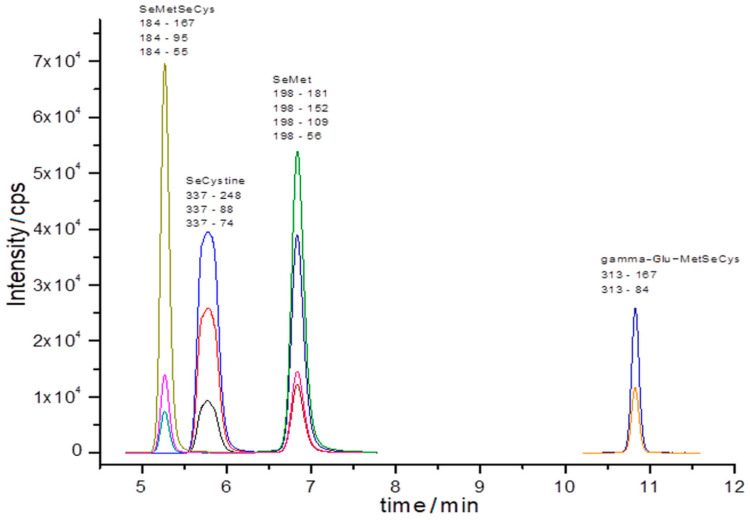
MRM chromatogram registered for standards solution (transitions precursor ion → product ion) of 250 µg/L of each compound: SeMetSeCys, SeCys, SeMet, and gamma-Glu-MetSeCys.

The sample chromatogram obtained with ultra-high performance liquid chromatography electrospray ionization (UHPLC-ESI)-QqQ-MS/MS for selenium standards containing 250 µg/L Se each (SeMet, SeMetSeCys, SeCys, and gamma-Glu-MetSeCys) is shown in Figure 3.

**Figure 4:**
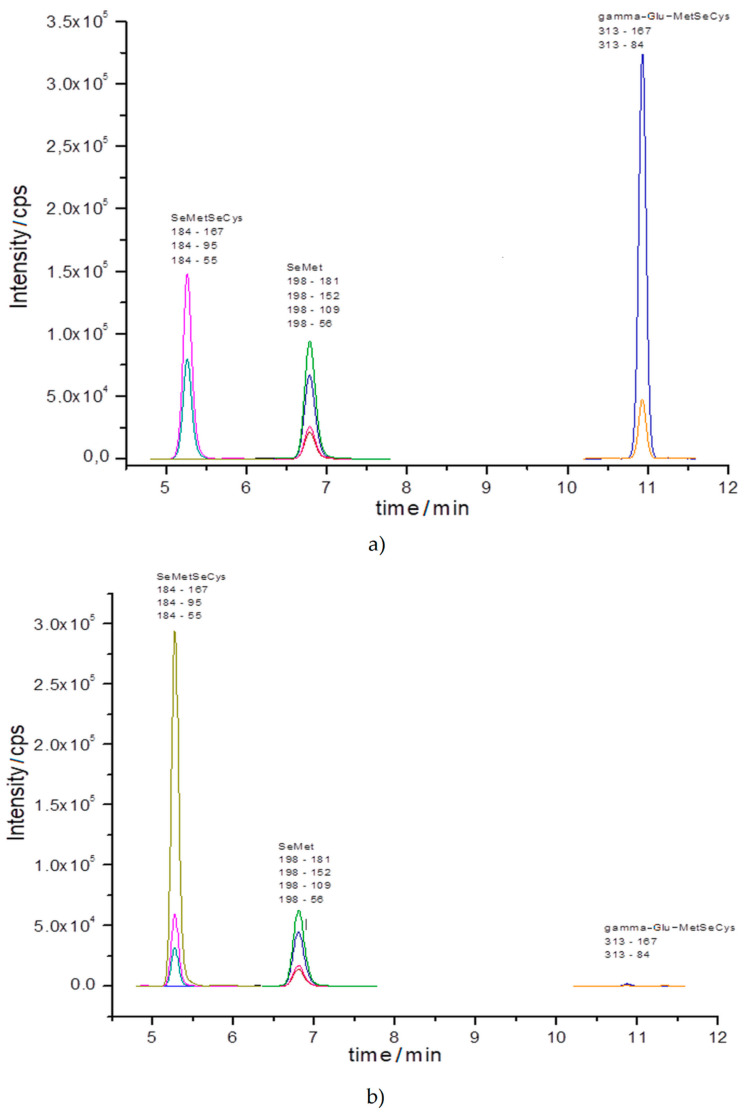

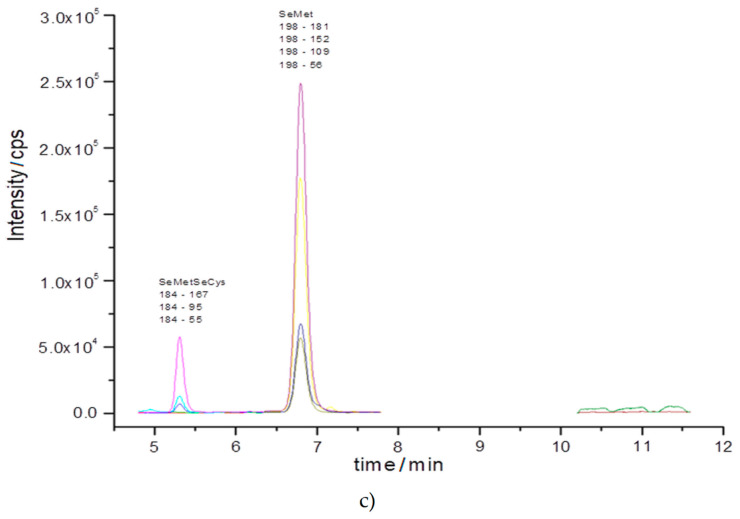
MRM chromatogram registered for extracts of (**a**) onion, (**b**) radish, and (**c**) sunflower sprouts grown in 60 mg Se/L medium (transitions precursor ion → product ion) by UHPLC-ESI-QqQ-MS/MS.

Chromatograms registered for extracts from sprouts are presented in Figure 4a–c and respectively.

In all cases, signals for MRM transitions unique for SeMet and SeMetSeCys are visible, thus confirming their presence in all types of sprouts. Surprisingly, the unique signals for MRM transitions for gamma-Glu-MetSeCys were only registered for the onion sprouts, but not radish or sunflower sprouts, despite being detected by HPLC-ICP-MS. As the electrospray ionization suffers from the ion suppression in the presence of complex matrix, this could decrease the detection power of this technique, leading to the lack of the signals of MRM unique transitions for gamma-Glu-MetSeCys. Unfortunately, no MRM signals characteristic for SeCys was registered by UHPLC-ESI-QqQ-MS/MS in the analyzed samples.

#### 2.2.3. Identification of Selenium Compounds with the use of UHPLC-ESI-Orbitrap-MS/MS

In order to search for those selenium compounds which were identified neither by HPLC-ICP-MS nor by UHPLC-ESI-QqQ-MS/MS, an electrospray mass ionization spectrometer equipped with a high-resolution mass analyzer Orbitrap (ESI-Orbitrap-MS/MS) coupled to an ultra-high performance liquid chromatography (UHPLC) was used. Considering the capability of Orbitrap mass analyzer to measure *m*/*z* with an accuracy below 1 mg/kg, its high resolving power up to 500,000, and its dynamic range over several orders of magnitude, its use is indispensable in screening analyses of unknown species in complex matrices of biological samples. The detailed investigation was focused on all types of sprouts, however for the clarity of presentation, the results will be given using radish sprouts as an example. This part of the study aimed to search for non-identified seleno-compounds.

To decrease possible matrix effects all extracts were preliminarily fractionated with the use of ion-exchange chromatography. Those fractions containing seleno-compounds were selected with the use of ICP-MS by monitoring ^82^Se isotope. In the case of radish sprouts, all together 14 fractions were chosen for further examination: nine main fractions (RF1 ÷ RF9) and several sub-fractions of RF2 (a, b, c) and RF3 (a, b, b, c). The summary of results obtained for radish sprouts is given in Table 3, containing retention times for collected fractions defined by HPLC-ICP-MS, retention times for UHPLC-ESI-Orbitrap-MS/MS, as well as numerical data of *m*/*z* for identified ions, both theoretical and experimental. Fractions were isolated directly from the HPLC column after previous HPLC-ICP-MS detection.

For SeMetSeCys a high in-source fragmentation was observed under applied electrospray ionization conditions. This phenomenon has already been reported in the literature [29,49]. Despite the attempt of optimization of ESI conditions, we did not manage to eliminate in-source fragmentation of SeMetSeCys. However, this does not interfere with identification analysis, since in each case MS/MS spectrum was recorded and evaluated confirming the identity of SeMetSeCys both in standard samples and real vegetable extract samples.

**Figure 5:**
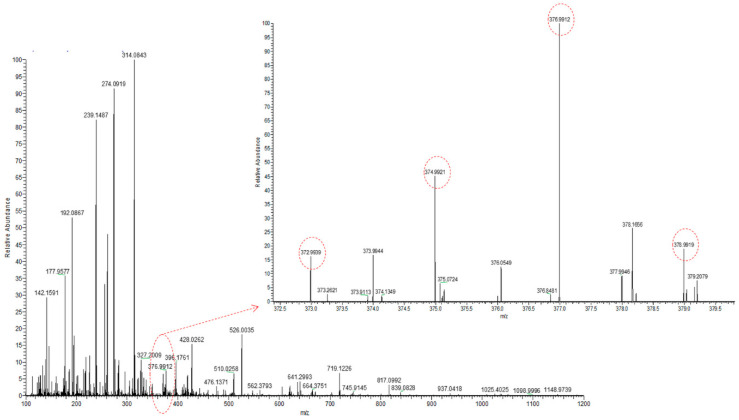
MS spectrum registered for the ion of 2,3-DHP-selenocysteine-cysteine with *m*/*z* 376.9912, with isotopic pattern characteristic for selenium compounds.

Continuing the sustainable characterization of selenium species, the presence of already identified SeMetSeCys and SeMet was confirmed. As an example, for RF6 (Figure 5), an isotopic pattern of the pseudo-molecular ion with *m*/*z* equal to 376.9912 was registered, which could be assigned to 2,3-DHP-selenocysteine-cysteine (theoretical *m*/*z* is equal to 376.9916) and corresponds to an unknown compound U6 (HPLC-ICP-MS in Figure 2).

The similar spectrum was registered for other compounds being present in some of the fractions. On the MS spectra obtained for SeMet found in RF3, pseudo-molecular ions, *m*/*z* 198.0027 and *m*/*z* 180.9764 (with H_2_O neutral loss), were registered. Finally, gamma-Glu-MetSeCys was found in RF8. Interestingly, a signal corresponding to *m*/*z* equal to 332.0242 (Figure S1) was also identified in RF1, which was assigned to 5′-seleno-adenosine with theoretical *m*/*z* equal to 332.0256. Thus, it can be assigned as an unknown compound named U4 on HPLC-ICP-MS chromatogram (Figure 2b).

In RF4, a compound with *m*/*z* ratio equal to 345.0193 was registered, to which 2,3-DHP-selenolanthionine was assigned (with theoretical *m*/*z* ratio equal to 345.0193 (Figure S2) corresponding to unknown compound U5. This compound was already described in the literature as one of the selenium metabolites in the yeast [14,16]. Next, in RF5, a conjugate of Se-S-cysteineselenoglutathione with theoretical *m*/*z* ratio of the pseudo-molecular ion equal to 475.0393 (Figure S3) was also identified.

Furthermore, with the results of the detailed investigation of the sunflower sprouts (not shown here), those with the highest relative content of unknown compounds confirm the presence of the following seleno-molecules: glutathione-2,3-DHP-selenocysteine (*m*/*z* = 563.0551) [53]; 2,3-DHP-selenocysteine-cysteinealanine (*m*/*z* = 448.0281) [47]; glutamyl-glycinyl-N-2,3-dihydroxy-propionyl-selenocysteine (*m*/*z* = 434.0130) [16]; 2,3-DHP-selenolanthionine (*m*/*z* = 345.0196); Se-S conjugate of cysteine-selenoglutathione (*m*/*z* = 475.0396); 2,3-DHP-selenocysteine-cysteine (*m*/*z* = 376.9916). By using UHPLC-ESI-Orbitrap-MS/MS the presence of gamma-Glu-MetSeCys (*m*/*z* = 313.0297) and SeMet (*m*/*z* = 198.0028) in sunflower extracts was confirmed. The same compounds were identified in samples of onion sprouts.

## 3. Materials and Methods

### 3.1. Preparation of Sprout’s Samples and Investigation of Selenium Intake

#### 3.1.1. Se-Enriched Sprouts

Sunflower (*Helianthus annuus*), onion (*Allium cepa*), and radish (*Raphanus sativus*) seeds were purchased from a Seedman’s Shop in Warsaw, Poland.

For the germination of seeds, tap water (for control samples) or tap water enriched with sodium selenite (for test samples) was used. The enriched solutions contained: 10 mg/L, 20 mg/L, 40 mg/L, and 60 mg/L of selenium, respectively. After harvesting, all plants were air-dried below 40 °C, grounded and stored at 4 °C.

Lyophilizer Alpha 1-2 LD plus (Martin Christ, Osterode, Germany) was used for the lyophilization of fresh sprout tissues.

#### 3.1.2. Determination of Total Selenium in Sprouts

Approximately 0.25 g of lyophilized sprouts were digested with 5 mL of HNO_3_ (65%, *v*/*v*). A microwave digestion system (Milestone UltraWave, Sorisole, BG, Italy) with Teflon vessels was used. The heating program was as follows: (i) 500 W/10 min; (ii) 1000 W/15 min; (iii) cooling/5 min. Transparent solutions were transferred into plastic tubes, diluted with deionized water to 20 mL, and stored in a refrigerator at 4 °C before measurements. Samples were diluted an additional 6 times before measurement.

Selenium content was determined by ICP-MS monitoring ^82^Se isotope. External calibration was performed with the standard solutions containing 1 μg/L, 10 μg/L, and 100 μg/L of selenium standard.

The validation of the analytical procedure was performed by analyzing a certified reference material, BCR-CRM 402, white clover, with the certified value for selenium (6.70 mg/kg ± 0.25 mg/k). The obtained results (6.74 mg/kg ± 1.01) mg/kg are considered as being within the uncertainty of the certified value.

#### 3.1.3. Extraction of Selenium Species from Sprouts

For 0.1 g of dry weight of lyophilized sprouts, 5 mL of a solution containing 25 mg of protease and 25 mg of lipase in water were used. Aliquots were incubated in a thermostat equipped with a magnetic stirring device at 37 °C ± 1 °C for 8 h. The supernatants were separated from the solution by centrifugation for 30 min at 7500× *g* and then were filtered through 0.45 µm membrane filters. The supernatant was stored in the fridge after the addition of 5 μL β-mercaptoethanol (0.1%) to avoid oxidation. Next, the supernatants were stored at −20 °C.

### 3.2. Instrumentation

An inductively coupled plasma mass spectrometer (NexION 300D ICP Mass Spectrometer, Perkin Elmer SCIEX, Norwalk, CT, USA) was used. A conventional Meinhard nebulizer and a quartz cyclonic spray chamber were used for sample introduction. The ICP-MS conditions were as follows: plasma power 1100 W, plasma argon flow 15 L/min, auxiliary gas flow 1.21 L/min, nebulizer gas flow 0.86 L/min. Determination of selenium by ICP-MS was performed in a standard system without a collision and reaction chamber. The validation parameters of procedure determination of selenium by ICP-MS technique in the standard mode confirmed its validity. In order to evaluate the analytical performance of ICP-MS and assure the validity of results, the selection of experimental parameters of the entire analytical procedure as well as selection of monitored isotope, certified reference materials (e.g., BCR-CRM 184, BCR-402, and SELM-1) were used. The selection of monitored isotope by argon ICP-MS was based at first on analyzing possible interferences arising from other species, then it was checked by analyzing selected CRMs. Only the results calculated with the signal monitored for ^82^Se isotope were within the limit of uncertainty given for the certified value. Therefore, during the examination of the total selenium content, the ^82^Se isotope was used.

#### 3.2.1. Limit of Quantification and Limit of Detection

Limit of quantification (LOQ) and limit of detection (LOD) were verified by measuring the analytical signal of three blank samples. Such samples were obtained by mixing all reagents used during sample preparation and executing exactly the same decomposition program that was used in the case of analytical samples. Limit of quantification was calculated using Equation (1).

LOQ = X + 10α(1)

Limit of detection was calculated using Equation (2).

LOQ = X + 6α(2)

where X is a mean value for analytical signal (concentration) and α is its standard deviation.

#### 3.2.2. Precision

Precision was investigated by monitoring relative standard deviation RSD for six independent samples enriched with selenium. Each sample was measured five times. Precision was less than 3% for an assay.

HPLC (Agilent 1290 Infinity system, Agilent Technologies, Wood Dale, IL, USA) was coupled to the ICP-MS (Elan 6100 DRC ICP-MS, Perkin Elmer SCIEX, Norwalk, CT, USA).

The chromatographic column type Hamilton PRP X100 (250 mm × 4.6 mm, particle size 10 μm) from Hamilton (USA) was used. As eluent, a 5 mM ammonium acetate buffer (pH 4.7) and B100 mM ammonium acetate buffer (pH 4.7) were applied. The mobile phase was delivered at 1.0 mL/min in the following gradient mode: (0–4) min, 100% A; (4–7) min, from 0% B to 100% B; (7–30) min, 100% B. The injection time was 100 μL. Using an Agilent 1290 Infinity system (Agilent Technologies, Wood Dale, IL, USA) (coupled to Agilent 6460 Triple Quadrupole mass spectrometer (Agilent Technologies, Wood Dale, IL, USA) equipped with electrospray ionization source (ESI) operating in positive ion mode. ESI conditions were as follows: nebulizing gas temperature, 300 °C; nebulizing gas flow, 10 L/min; sheath gas temperature, 200 °C; sheath gas flow, 11 L/min; negative ionization mode source voltage, 3.7 kV. Nitrogen was used as a nebulizing and sheath gas. The mass spectrometer was operated in the multiple reaction monitoring (MRM) mode following optimization of working conditions (fragmentor voltage, F, and collision energy, CE) for seleno-compounds using a standard solution at the concentration of 1 mg/L (in acetonitrile/water 50%/50% (*v/v*) solution) in the direct infusion mode. Seleno-compounds were separated on HyperCarb column (Thermo Scientific, Liverpool, NY, USA) (150 mm × 4.6 mm, particle size 5 µm) at 35 °C. Water with 0.1% formic acid and acetonitrile with 0.1% formic acid were used as eluent A and B, respectively. The following gradient elution was applied: (0–2) min, 3% B; (2–16) min, linear increase from 3% B to 50% B; (16–17) min, 50% B; (17–19) min, linear increase from 50% B to 90% B; (19–24), 3% B. The injection volume was 20 µL. Total time of chromatographic separation was 24 min.

UHPLC-ESI-Orbitrap-MS/MS: Measurements were conducted using an Agilent 1290 UHPLC (Agilent Technologies, Wood Dale, IL, USA) coupled to an Orbitrap Fusion Tribrid Mass Spectrometer (Thermo Fisher Scientific, Liverpool, NY, USA). A HyperCarb column (150 mm × 4.6 mm) with 5 µm particle size (Thermo Scientific, Liverpool, NY, USA) was used. The injection volume was 20 µL. Compounds were eluted from the column at the flow rate of 0.5 mL min^−1^ with the increasing linear gradient from 3–50% of solvent B (acetonitrile with 0.1% formic acid); 0.1% aqueous formic acid was used as solvent A. The eluted compounds were ionized in the positive ion mode with a capillary voltage of 3.9 kV in the heated electrospray ion source (HESI). The working ion source parameters, optimized on the total ion current (TIC) values, were as follows: sheath gas flow, 40 L min^−1^; auxiliary gas flow, 15 L min^−1^; ion transfer tube temperature, 350 °C; and vaporizer temperature, 300 °C. Survey scans were recorded with the Orbitrap mass analyzer at a resolving power of 60.000 in the *m*/*z* range of 100–1200 and from each survey scan the top 10 most abundant singly charged ions (parent ions) were fragmented by higher-energy collision induced dissociation (HCD). The precursor ion was excluded from further fragmentation for 60 s. The product ions were analyzed in the Orbitrap analyzer at the resolving power of 30.000. Survey scans were searched manually using Xcalibur 3.0 (Thermo Scientific, Liverpool, NY, USA) for the presence of isotopic pattern characteristic for selenium-containing compounds. MS/MS spectra were interpreted manually to elucidate the structure of the unknown compound.

All procedures used with this work were validated and the obtained analytical parameters were evaluated as to fit the aim of conducted investigation.

### 3.3. Reagents

All chemicals were of analytical grade and used without further purification. Nitric acid (V) 65%, hydrochloric acid 37%, and multi-elemental ICP-MS standard solutions were obtained from Merck (Germany). Water for analysis was obtained from Milli-Q system (Millipore, Billerica, MA, USA). Lipase from *Pseudomonas cepacia* and protease from *Streptomyces griseus* were obtained from Aldrich Chemical Company Inc. (Saint Louis, Mo, USA).

Sodium selenite (Na_2_SeO_3_) and sodium selenate (Na_2_SeO_4_) were obtained from Sigma (Darmstadt, Germany). Selenomethionine (SeMet), SeMetSeCys, and selenocystine (SeCys), were purchased from Sigma (Saint Louis, Mo, USA). L-γ-glutamyl-Se-methylseleno-L-cysteine (gamma-Glu-MetSeCys) was obtained from former PharmaSe Inc. (Lubbock, TX, USA). Sodium dodecyl sulfate (SDS), tris(hydroxymethyl) aminomethane (TRIS), and sodium hydroxide were obtained from Sigma (Saint Louis, Mo, USA). Peroxide of hydrogen 30% and ammonium acetate were purchased from POCh (Gliwice, Poland). TES solution was composed of TRIS (Sigma, Saint Louis, Mo, USA), ethylenediaminetetraacetic acid (EDTA) (POCH, Gliwice, Poland), and saccharose (Sigma, Saint Louis, Mo, USA).

A reference material BCR-CRM 184, bovine muscle with a total selenium content of 0.183 mg/kg (±7%), was obtained from European Commission—Joint Research Centre, Institute for Reference Materials and Measurements (Geel, Belgium).

A reference material BCR-402, white clover with total selenium content of 6.70 (±0.25) µg/g was from European Commission—Joint Research Centre, Institute for Reference Materials and Measurements (Belgium).

A reference material SELM-1, selenium enriched yeast, with certified value for SeMET 3190 ± 260 mg/kg was from National Research Council of Canada (NRC-CNRC).

## 4. Conclusions

The aim of the described project was to optimize the analytical methodology towards the determination of selenium as well as identify and characterize low-molecular-weight seleno-compounds in sprouts. A comprehensive procedure combining four analytical techniques based on mass spectrometry was developed to collect complementary information to characterize selenium speciation in plants. ICP-MS was used to determine the presence of selenium in entire sprouts, in extract solutions as well as in various chromatographic fractions. Then, HPLC-ICP-MS was applied to identify selected inorganic and organic seleno-compounds, with the advantages of being very sensitive towards selenium, but limited by available selenium standard compounds. Both UHPLC-ESI-QqQ-MS/MS and UHPLC-ESI-Orbitrap-MS/MS were used for the confirmation of the presence of several compounds of selenium in sprouts’ extracts and for the further identification of several unknown compounds, respectively. UHPLC-ESI-QqQ-MS/MS was found to be very useful for targeted analysis towards confirmation of the presence of selected seleno-molecules, however, it was limited by its sensitivity mainly due to the influence of matrix on ionization efficiency. Thus, the non-target approach using UHPLC-ESI-Orbitrap-MS/MS enables the final confirmation of preliminary detected compounds as well as the identification of a number of unknown selenium species for which the standards are not available.

The presented analytical protocol was found to be appropriate for conducting a complete characterization of the selenium speciation, so to evaluate the possible mechanism of biotransformation of selenium inorganic species to their organic derivatives in plants. It can serve as a tool to anticipate the usefulness of specific plants or its sprouts in being a safe source of low molecular weight seleno-compounds.

Although all measurement techniques used in this work were explored by others, the added value of the developed protocol is a comprehensive approach to characterize the speciation of selenium in plants. Moreover, we also explored new varieties of plants.

## Figures and Tables

**Table 1:** Total selenium concentration after 7 days of germination of sprout’s in solution containing selenium (Se) as selenite (Na_2_SeO_3_). The results are given as a mean value for *n* = 6 with uncertainty ±20% (k = 2).

Selenium in Nutrient Solution (mg/L Se)	Total Se Content (mg/kg Dried Weight) *		
	Sunflower Sprouts	Radish Sprouts	Onion Sprouts
**control sample**	<LOQ	<LOQ	1.51 ± 0.08
**10**	20 ± 1	64 ± 3	91 ± 5
**20**	46 ± 2	145 ± 7	198 ± 10
**40**	87 ± 4	304 ± 15	574 ± 29
**60**	132 ± 6	388 ± 19	825 ± 41

* Limit of quantification (LOQ) for Se 0.480 mg/kg.

**Table 2:** Optimized multiple reaction monitoring (MRM) QqQ-MS/MS working parameters for selenium compounds.

Compound	MRM Transition Precursor Ion *m*/*z* → Product Ion *m*/*z*	Fragmentor (V)	Collision Energy (V)
**SeMet**	198 → 181	80	5
	198 → 152	80	9
	198 → 109	80	25
	198 → 56	80	21
**SeMetSeCys**	184 → 167	50	5
	184 → 95	50	29
	184 → 55	50	21
**SeCys**	337 → 248	80	9
	337 → 88	80	21
	337 → 74	80	29
**gamma-Glu-MetSeCys**	313 → 167	70	13
	313 → 84	100	29

**Table 3:** Selenium compounds identified in the extract of radish sprouts enriched in selenium.

Sample/Fraction	t_R_ *		Seleno-Compound Identified by HPLC-ICP-MS	*m*/*z*_teor_	*m*/*z*_exp_	Chemical Formula (M+H^+^)	Seleno-Compound Identified by UHPLC-ESI-Orbitrap-MS/MS	References
	HPLC-ICP-MS	UHPLC-ESI-Orbitrap-MS/MS						
RF1	1.9–2.5	7.84	**U4**	332.0256	332.0242	**C_10_H_14_O_3_N_5_Se^+^**	**5′-seleno adenosine**	[49]
RF2a	2.5–3.0	5.17	**SeMetCys**	166.9606	166.9607	**C_4_H_7_O_2_Se^+^**	**SeMetCys –NH_3_**	[29]
RF2b	3.0–3.4	5.17		166.9606	166.9607	**C_4_H_7_O_2_Se^+^**	**SeMetCys–NH_3_**	[49]
RF2c	3.4–3.8	5.17		166.9606	166.9607	**C_4_H_7_O_2_Se^+^**	**SeMetCys–NH_3_**	
RF3a	4.0–4.3						**SeMet**	[50]
RF3b	4.3–5.0	6.57	**SeMet**	198.0028	198.0027	**C_5_H_12_O_2_NSe^+^**	**SeMet**	[16,51,52]
RF3b	4.3–5.0	6.57		180.9762	180.9764	**C_5_H_9_O_2_Se^+^**	**SeMet-NH_3_**	[49]
RF3c	5.0–5.7							
RF4	6.0–7.8	10.49	**U5**	345.0196	345.0193	**C_9_H_17_O_7_N_2_Se^+^**	**2,3-DHP-selenolanthionine**	[14,16]
RF5	7.8–8.9	10.05	**Se (IV)**	475.0396	475.0393	**C_13_H_23_O_8_N_4_SSe^+^**	**Se-S conjugate of cysteino-selenoglutathione**	[16,51]
RF6	8.9–9.8	12.05	**U6**	376.9916	376.9912	**C_9_H_17_O_7_N_2_SSe^+^**	**2,3-DHP-selenocysteine-cysteine**	[16,51]
RF7	9.8–10.8		**U7**					
RF8	10.8–11,6	11.22	**gamma-Glu-MetSeCys**	313.0297	313.0292	**C_9_H_17_O_5_N_2_Se^+^**	**gamma-Glu-MetSeCys**	[12,16]
RF9	11.6–13.0		**U8**					

* t_R_–retention time.

## Supplementary Materials

The following are available online at https://www.mdpi.com/1420-3049/25/12/2870/s1, Figure S1: title, Table S1: title, Video S1: title. Figure S1. MS spectrum registered for the ion of 5′-seleno adenosine with *m*/*z* 332.0242, with isotopic pattern characteristic for selenium compounds. Figure S2. MS spectrum registered for the ion of 2,3-DHP-selenolanthionine with *m*/*z* 345.0193, with isotopic pattern characteristic for selenium compounds. Figure S3. MS spectrum registered for the ion of Se-S conjugate of cysteino-selenoglutathione with *m*/*z* 475.0393, with isotopic pattern characteristic for selenium compounds.
