# Supplementary material for: Searching for Low Molecular Weight Seleno-Compounds in Sprouts by Mass Spectrometry
Source: Molecules. 2020 Jun 22;25(12):2870. doi: 10.3390/molecules25122870 (PMC7355765; doi:10.3390/molecules25122870)
Supplement: Supplementary file 1 [file molecules-25-02870-s001.pdf]

molecules

MDPI

Supplementary Materials

Searching for Low Molecular Weight Seleno-Compounds in Sprouts by Mass Spectrometry

Eliza Kurek, Magdalena Michalska-Kacymirow, Anna Konopka, Olga Kościuczuk, Anna Tomiak and Ewa Bulska *

Faculty of Chemistry, Biological and Chemical Research Centre, University of Warsaw, Żwirki i Wigury 101, 02-089 Warsaw, Poland; ekurek@cnbc.uw.edu.pl (E.K.); m.kacymirow@cnbc.uw.edu.pl (M.M.-K.); a.konopka@cnbc.uw.edu.pl (A.K.); o.kosciuczuk@cnbc.uw.edu.pl (O.K.); atomiak@cnbc.uw.edu.pl (A.T.)
* Correspondence: ebulska@chem.uw.edu.pl

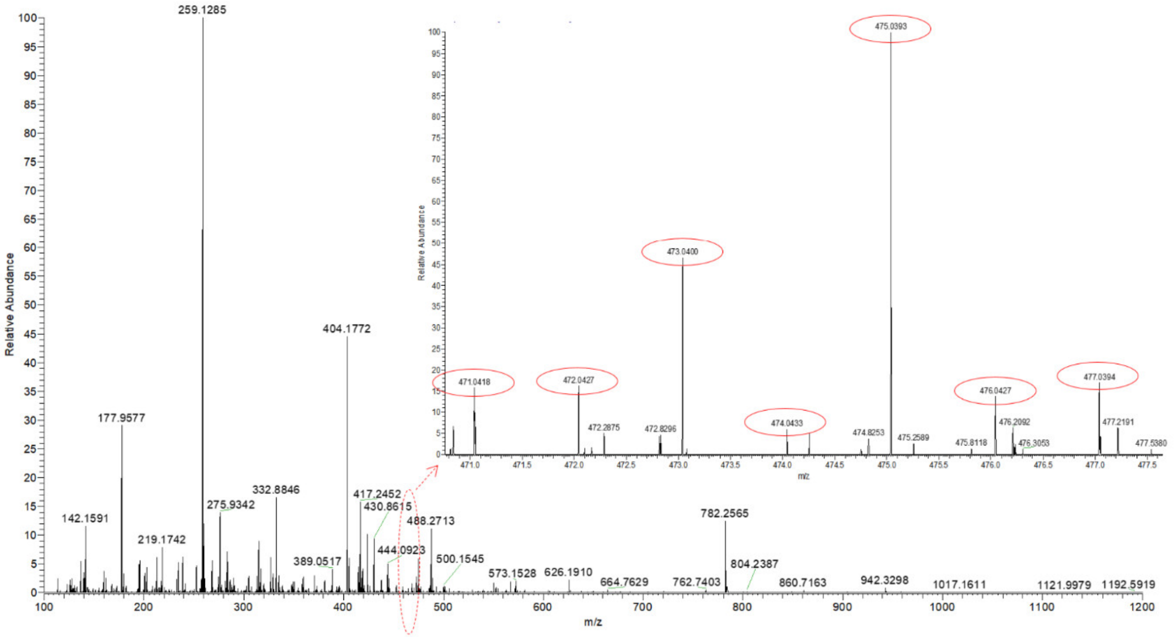

Figure S1. MS spectrum registered for the ion of 5'-seleno adenosine with m/z 332.0242, with isotopic pattern characteristic for selenium compounds.

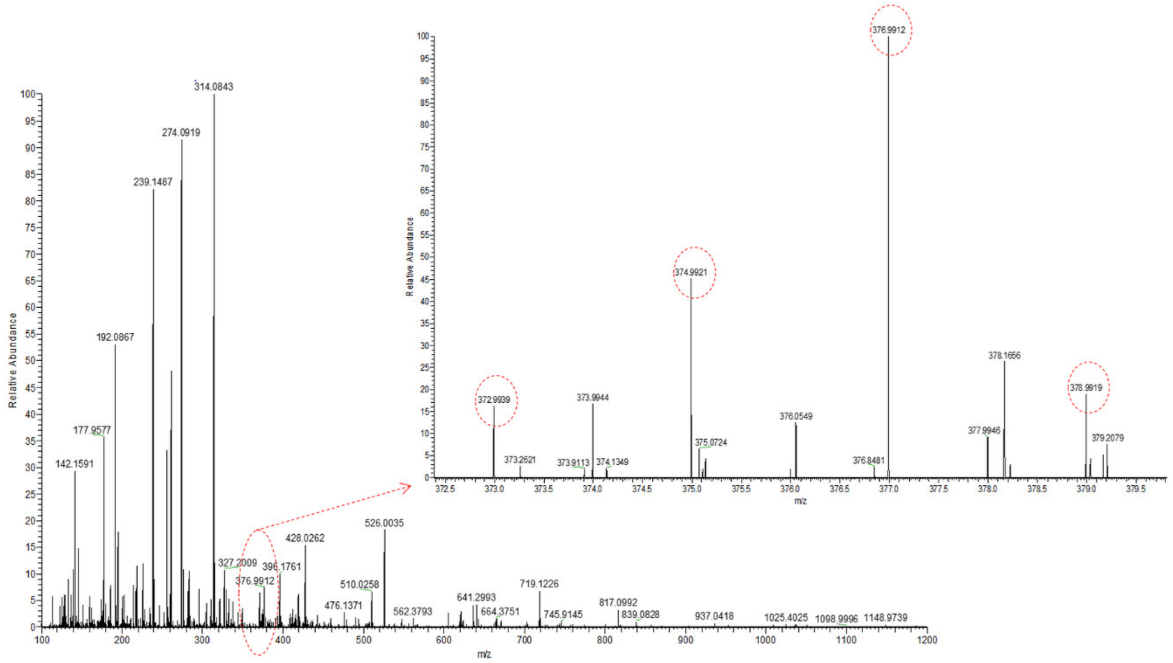

Figure S2. MS spectrum registered for the ion of 2,3-DHP-selenolanthionine with m/z 345.0193, with isotopic pattern characteristic for selenium compounds.

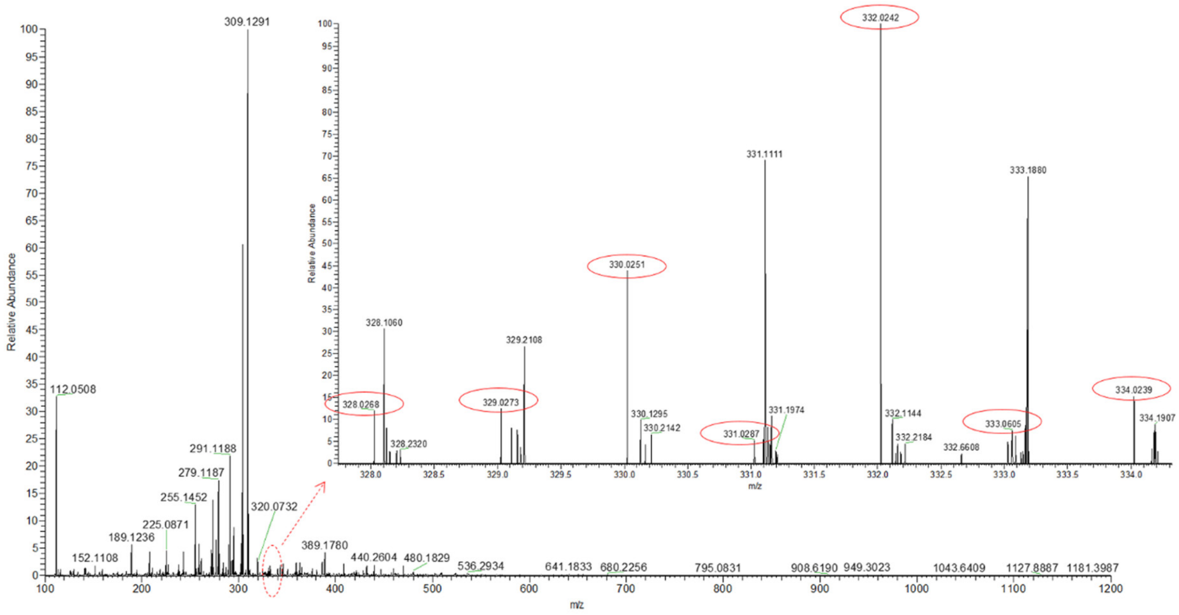

Figure S3. MS spectrum registered for the ion of Se-S conjugate of cysteino-selenoglutathione with m/z 475.0393, with isotopic pattern characteristic for selenium compounds.
